# Response to comment on “Effectiveness and safety of stem cell therapy for diabetic foot: a meta-analysis update”

**DOI:** 10.1186/s13287-023-03609-9

**Published:** 2024-03-22

**Authors:** Yuming Sun, Jinhong Zhao, Lifang Zhang, Zhexuan Li, Shaorong Lei

**Affiliations:** 1 Department of Plastic and Cosmetic Surgery, Xiangya Hospital, Central South University, 87 Xiangya Road, Changsha, 410008 Hunan Province China; 2 School of Health Policy and Management, Chinese Academy of Medical Sciences & Peking Union Medical College, Beijing, 100730 China

To the editor

We appreciate the opportunity to reply the comments on our article that concluded through meta-analysis update that stem cell therapy is effective in treating diabetic foot. These comments reflect heightened concerns about the effects of stem cell therapy in patients with diabetic foot. Based on their comments, we present discussions that will be more comprehensible to the readers.

Firstly, we apologize for any misunderstandings caused by the labels in Figs. 3, 5, 7, 8, S1, and S3 in the article [1]. However, we do not agree with the comment about “a fatal error lies in the authors’ lack of awareness about the Revman software.” In Revman’s forest plot, when the summary diamond falls on the right side of the null line, it means that the experimental group has more events than the control group, while falling to the left is the opposite. Therefore, it is necessary to consider whether the events that occur are protective or harmful. For example, in Fig. 3, the events in both groups are healing rates as protective events, while in the labels of the figure, we did not label “favors,” the summary diamond falls on the “Control[control]” side, which only indicating that more events occurred in the experimental group after summary. This also proves that stem cell therapy for diabetic foot is effective, which is consistent with the explanation in our article. Similarly, the same explanation is given to other figures.

Secondly, as the comment mentioned, “when incorporating a ‘zero event’ trial, the constant continuity correction method should be applied by adding a correction factor of 0.5 in case of zero events in one group.” However, due to the artificial addition of fictitious samples, continuity correction may lead to a large deviation in parameter estimation, especially when the two groups of samples are not balanced [2–4]. In our article, we adopt the Mantel–Haenszel method for “zero events,” which enables nearly unbiased estimates [3]. Thirdly, thanks for the comments on the aesthetics of our forest plot. As we all know, the summarized diamond of the forest plot is only the direction of the summarized effect values visually, and the specific results also depend on the effect values in the figure, which does not affect the interpretation of the results in our figures. We will also pay attention to this problem in the subsequent research. Fourthly, the authors recommend us to register with the International Prospective Register of Systematic Reviews (PROSPERO) or the International Platform of Registered Systematic Review and Meta-analysis Protocols (INPLASY) before conducting a systematic review and meta-analysis. Thanks for the author’s kind reminder. We should register before conducting a systematic review and meta-analysis, but we did not do so because we were updating a previously published meta-analysis [5].

In summary, we appreciate the commentators’ interest in our research which indicate Stem Cell Research and Therapy is a high-quality journal. Although the comments raised did not affect the conclusion of our article, our discussion made our research more understandable to readers.

We hope, we have clarified the queries to the best extent possible.

## References

[1] Sun Y, Zhao J, Zhang L, Li Z, Lei S (2022). Effectiveness and safety of stem cell therapy for diabetic foot: a meta-analysis update. Stem Cell Res Ther.

[2] Böhning D, Mylona K, Kimber A (2015). Meta-analysis of clinical trials with rare events. Biom J.

[3] Günhan BK, Röver C, Friede T (2019). Random-effects meta-analysis of few studies involving rare events. Res Synth. Methods.

[4] Kuss O (2015). Statistical methods for meta-analyses including information from studies without any events-add nothing to nothing and succeed nevertheless. Stat Med.

[5] Guo J, Dardik A, Fang K, Huang R, Gu Y (2017). Meta-analysis on the treatment of diabetic foot ulcers with autologous stem cells. Stem Cell Res Ther.

